# Glis family proteins are differentially implicated in the cellular reprogramming of human somatic cells

**DOI:** 10.18632/oncotarget.20334

**Published:** 2017-08-18

**Authors:** Seo-Young Lee, Hye Bin Noh, Hyeong-Taek Kim, Kang-In Lee, Dong-Youn Hwang

**Affiliations:** ^1^ Department of Biomedical Science, College of Life Science, CHA University, Seongnamsi, Gyeonggido 13488, Korea

**Keywords:** somatic cells, induced pluripotent stem cells, glis family proteins, cellular reprogramming, adipose derived stromal cells

## Abstract

The ground-breaking discovery of the reprogramming of somatic cells into pluripotent cells, termed induced pluripotent stem cells (iPSCs), was accomplished by delivering 4 transcription factors, Oct4, Sox2, Klf4, and c-Myc, into fibroblasts. Since then, several efforts have attempted to unveil other factors that are directly implicated in or might enhance reprogramming. Importantly, a number of transcription factors are reported to retain reprogramming activity. A previous study suggested Gli-similar 1 (Glis1) as a factor that enhances the reprogramming of fibroblasts during iPSC generation. However, the implication of other Glis members, including Glis2 and Glis3 (variants 1 and 2), in cellular reprogramming remains unknown.

In this study, we investigated the potential involvement of human Glis family proteins, including hGlis1-3, in cellular reprogramming. Our results demonstrate that hGlis1, which is reported to reprogram human fibroblasts, promotes the reprogramming of human adipose-derived stromal cells (hADSCs), indicating that the reprogramming activity of Glis1 is not cell type-specific. Strikingly, hGlis3 promoted the reprogramming of hADSCs as efficiently as hGlis1. On the contrary, hGlis2 showed a strong negative effect on reprogramming.

Together, our results reveal clear differences in the cellular reprogramming activity among Glis family members and provide valuable insight into the development of a new reprogramming strategy using Glis family proteins.

## INTRODUCTION

Since the ground-breaking discovery by Shinya Yamanaka, four transcription factors consisting of Oct4, Sox2, Klf4, and c-Myc (termed OSKM) have been widely used to generate induced pluripotent stem cells (iPSCs) [1, 2]. In addition, several reprogramming factors that either replace some of the Yamanaka factors or enhance their reprogramming efficiency have been reported, and the list of the alternative reprogramming factors continues to grow [3].

The Gli-similar (Glis) protein family is closely related to Gli proteins and constitutes a subfamily of the Krüppel-like zinc finger family, which is characterized by classical Cys_2_-His_2_ zinc fingers [4]. Glis family members are expressed in a spatial manner in mouse tissues. For example, mouse Glis1 (mGlis1) is expressed in the kidney, placenta, testis, thymus, brain, and colon [5]; mGlis2 is expressed in the kidney, intestine, brain, lung, prostate, and colon [6, 7]; and mGlis3 is expressed in the kidney, ovary, uterus, brain, pancreas, thymus, lung, and thyroid [8, 9]. The somewhat differential expression patterns among the mGlis family members suggest their distinct roles in certain types of tissues.

The functions of Glis proteins have mostly been studied using knockout mouse models. The Glis2-knockout mouse develops a type of kidney failure, termed nephronophthisis (NPHP), and the Glis3-null mouse develops polycystic kidney disease, diabetes, and hypothyroidism [10–14]. In contrast, thus far, no study has reported a Glis1-null mouse model. However, a recent study demonstrated that Glis1 markedly enhanced iPSC generation from fibroblasts [15, 16].

Intriguingly, a high degree of sequence homology is apparent among the DNA-binding domains of the mGlis proteins. For instance, mGlis1 is 93% and 58% identical in amino acid sequence in comparison to mGlis3 and 2, respectively [5, 8]. Therefore, it is tempting to speculate that some genes might be regulated by more than one member of the mGlis protein family.

The human Glis (hGlis) protein family is composed of hGlis1, hGlis2, and two variants of hGlis3 (hGlis3v1 and hGlis3v2) and contains five Cys2-His2 zinc finger motifs like the Gli family protein [12]. To date, direct sequence comparisons among hGlis family members have not been reported.

In this study, we show that the hGlis family proteins share significant sequence homology, especially in the zinc finger DNA-binding domain. Based on the high sequence homologies in the DNA-binding domain sequences, we investigated whether all hGlis family proteins share the ability to promote cellular reprogramming. Our results demonstrated that hGlis1 and hGlis3, but not hGlis2, enhanced human iPSC generation, indicating differential roles of the hGlis family proteins in cellular reprogramming.

## RESULTS

### Comparison of the amino acid sequences of the human Glis family proteins

**Figure 1:**
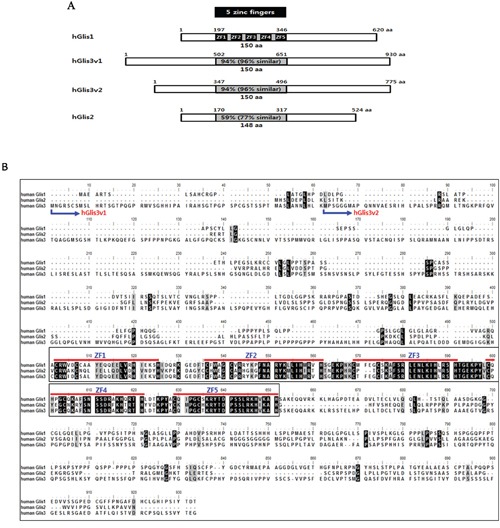
Sequence comparisons of the human Glis family proteins **(A)** The amino acid sequence homologies among hGlis proteins are shown in the 5 zinc finger-containing DNA-binding domains (grey box). The 5 black boxes in the DNA-binding domain of hGlis1 indicate zinc finger sequences. **(B)** The amino sequences of the hGlis proteins were aligned. Perfectly matched amino acids among hGlis1, 2 and 3 are represented by black boxes, and the amino acid positions with one mismatch among the hGlis proteins are marked in grey. The 5 red lines in the middle of the entire sequences indicate the 5 zinc finger sequences (ZF1-5). The N-terminal amino acids of the two variants of hGlis3 (hGlis3v1 and hGlis3v2) are marked at the beginning of the entire amino acid sequences.

The human Glis family consists of four proteins: hGlis1, hGlis2, and two variants of hGlis3 (hGlis3v1 and hGlis3v2). The two variants of hGlis3 are formed by different transcription initiation sites of the *Glis3* gene. The sizes of the hGlis family proteins vary; hGlis1 is composed of 620 amino acids (aa), hGlis2 is 524 aa, hGlis3 variant 1 is 930 aa, and hGlis3 variant 2 is 775 aa (Figure 1A). The DNA-binding domains of all hGlis proteins consist of 5 Cys_2_-His_2_ zinc finger domains (ZF1-5) (Figure 1B).

Intriguingly, the amino acid sequences of the zinc finger DNA-binding domains (150 aa) share high homology among the family members. The hGlis1 zinc finger DNA-binding domain shares 94%, 94%, and 59% homology with hGlis3v1, hGlis3v2, and hGlis2, respectively (Figure 1A, 1B). When we extended the sequence homology to the amino acids with similar properties, the homologies between the DNA-binding domains of hGlis1 and those of Glis3v1, hGlis3v2, and hGlis2 increased to 96%, 96%, and 77%, respectively (Figure 1A, 1B). However, the sequences other than the DNA-binding domain are quite diverse among the family members, and these sequences might determine the distinct regulatory properties of the hGlis family proteins (Figure 1B). The high sequence homologies of the zinc finger DNA-binding domains (approximately 150 aa) among hGlis family proteins suggest a potential overlap of some downstream genes regulated by the hGlis members. Furthermore, it is noticeable that the homology of total amino acid sequences between hGlis1 and hGlis3v2 is higher than that between hGlis1 and hGlis2 (Supplementary Figure 1).

### Examination of the reprogramming activities of the hGlis family proteins

Recently, hGlis1 was suggested to enhance the reprogramming of both mouse and human fibroblasts [15]. However, the role of other hGlis family proteins in reprogramming has not yet to be investigated. To examine the reprogramming activities of the hGlis family proteins, we transduced hADSCs with retroviruses expressing each *hGlis* gene along with a mixture of retroviruses expressing each of the four Yamanaka reprogramming factors, including Oct4, Sox2, Klf4, and c-Myc [1]. Semiquantitative RT-PCR analysis at 70 hours after retroviral transduction confirmed the overexpression of the exogenously introduced transcription factor genes in the hADSCs (Supplementary Figure 2). As previously reported [17], the expression of the transgenes was shut down, while the endogenous *Oct4, Sox2, Klf4*, and *c-Myc* genes were turned on after reprogramming (Supplementary Figure 3).

**Figure 2:**
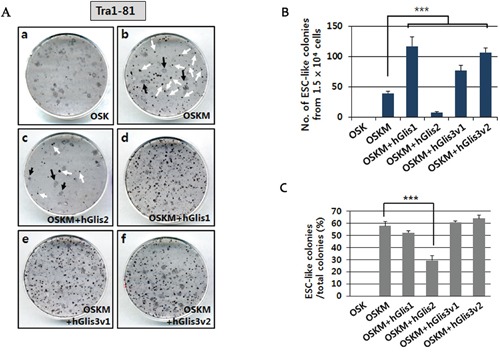
Comparison of the reprogramming activities of Glis proteins **(A)** The reprogramming efficiencies of the Glis proteins were compared by counting the number of ESC-like (iPSC) colonies generated after staining with the pluripotency marker Tra1-80. Immunostaining was performed at day 21 post-transduction of the hADSCs with the transgene-expressing retroviruses. Two different forms of colonies were detected, including ESC-like colonies with a clear boundary (i.e., white arrows in Figure 2A, b & c) and transformed cell colonies (i.e., black arrows in Figure 2A, b & c). **(B)** The number of ESC-like colonies formed from 1.5 × 10^4^ hADSCs is indicated in the graph. n = 3, ***p<0.001. **(C)** The percentage of ESC-like colonies among the total colonies (both ESC-like and transformed cell colonies) is shown in the graph. n = 3, ***p<0.001.

Our result showed a significant increase of iPSC generation when hGlis1 or hGlis3 was overexpressed in addition to the 4 Yamanaka factors (OSKM) (Figure 2A, 2B). Both variants of hGlis3 (hGlis3v1 and hGlis3v2) enhanced the reprogramming efficiency to a comparable level (Figure 2A e-f and 2B). Intriguingly, the deletion of the zinc finger DNA domain (hGlis3(ΔZFD)), the C-terminal amino acid sequences (hGlis3(ΔC)), and the N-terminal amino acid sequences (hGlis3(ΔN)) all reduced the reprogramming efficiency, suggesting that the whole sequence of hGlis3 is required for maximal activity (Supplementary Figure 4).

Strikingly, hGlis2 overexpression elicited a drastically decreased number of ESC-like colonies than that generated by the 4 Yamanaka factors only (Figure 2A b, c, and 2B). No ESC-like colonies were detected when c-Myc was omitted (i.e., using Oct4, Sox2, and Klf4 only (OSK)) for reprogramming, although the formation of many transformed cells was apparent (Figure 2A a and 2B). When hGlis1 was added to this set of genes (OSK), no ESC-like colonies were detected (data not shown), indicating that hGlis1 did not replace c-Myc in our reprogramming experimental conditions using hADSCs. This result is somewhat different from that of a previous study that used human dermal fibroblasts for reprogramming [15]. This discrepancy may be caused by difference in the experimental conditions including the primary cells used for iPSC generation (e.g., hADSC vs. human dermal fibroblasts).

The percentage of ESC-like colonies among the total colonies was not significantly changed when either hGlis1 or hGlis3 was added to the 4 Yamanaka factors (OSKM) (Figure 2C), suggesting that the addition of the Glis1 and Glis3 does not affect the fate determination into either fully reprogrammed or transformed cells. In contrast, hGlis2 overexpression drastically reduced this percentage, further supporting the notion that hGlis2 functions unfavorably in reprogramming (Figure 2C).

### Characterization of iPSCs generated in the presence of hGlis3

Glis3-iPSC lines generated by hGlis3v1, Oct4, Sox2, Klf4, and c-Myc were passaged over 15 times and were subjected to characterization for their properties as pluripotent stem cells. First, using a DNA fingerprinting analysis, we confirmed that the Glis3-iPSC lines were indeed derived from the hADSCs that were originally used for our iPSC generation (Supplementary Table 4).

**Figure 3:**
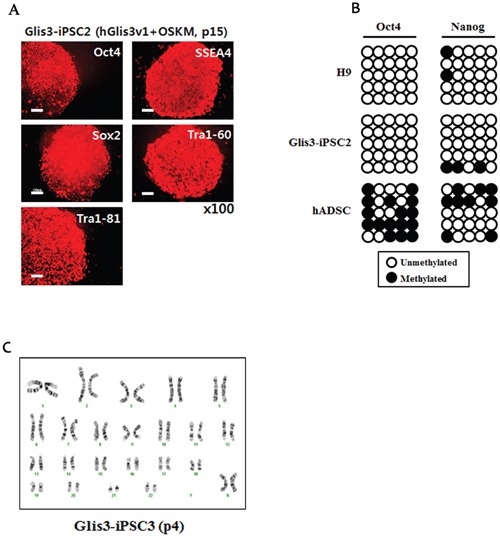
Characterization of Glis3-iPSCs generated by 4 Yamanaka factors and hGlis3v1 **(A)** Immunostaining of the Glis3-iPSC2 line (at passage 15) was performed using antibodies recognizing undifferentiated pluripotent cell markers, such as Oct4, SSEA4, Sox2, Tra1-60, and Tra1-81. Scale bar: 100 μm. **(B)** DNA methylation patterns of the promoters of Oct4 and Nanog were compared among H9-hESCs, the Glis3-iPSC2 cell line, and hADSCs. The black circles indicate methylated CpG sites, and the white circles show unmethylated CpG sites. **(C)** The presence of chromosome abnormalities in the Glis3-iPSC3 cell line was examined using G-banding analysis.

Immunostaining experiments showed that the iPSCs expressed hESC markers, such as Oct4, Sox2, SSEA4, Tra1-60, and Tra1-81 (Figure 3A). Like H9-hESCs, the Glis3-iPSC2 cell line retained a hypomethylation pattern in the promoter area of the *Oct4* and *Nanog* genes (Figure 3B). In contrast, the original primary cells from which the iPSCs were generated, the hADSCs (p4), were hypermethylated in CpG sequences within the upstream sequences of the *Oct4* and *Nanog* genes (Figure 3B, shown on the most bottom row). No chromosomal abnormalities were detected in all the Glis3-iPSC lines (Figure 3C and data not shown).

**Figure 4:**
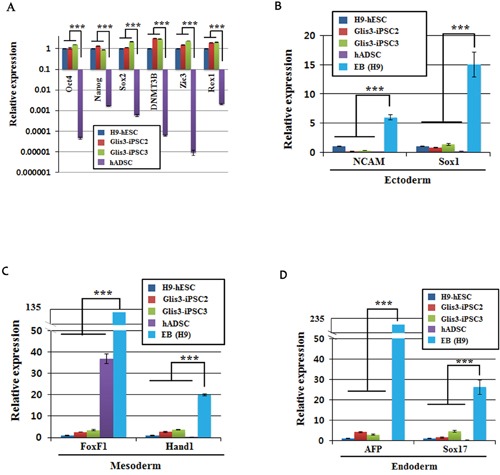
Quantitative RT-PCR analysis of Glis3-iPSCs The levels of mRNA expression in hESCs, the Glis3-iPSC2 and Glis3-iPSC3 cell lines, and hADSCs and the EB for undifferentiated pluripotent cell markers **(A)**, ectoderm markers **(B)**, mesoderm markers **(C)**, and endoderm markers **(D)** were examined using the primer pairs shown in Supplementary Table 1. The data were normalized using beta-actin mRNA. The expression of each gene in H9-hESCs was arbitrarily set at 1. Error bars, s.d.; n = 3. ***p<0.001.

A qRT-PCR analysis showed comparable expression levels of human pluripotent stem cell (hPSC) markers, such as Oct4, Nanog, Sox2, DNMT3B, Zic3, and Rex1, in hESCs, Glis3-iPSC2 cells, and Glis3-iPSC3 cells, while hADSCs displayed completely different expression patterns for the hPSC markers (Figure 4A). However, the two Glis3-iPSC lines did not express markers of the three germ layer cell derivatives (Figure 4B, 4C, and 4D).

**Figure 5:**
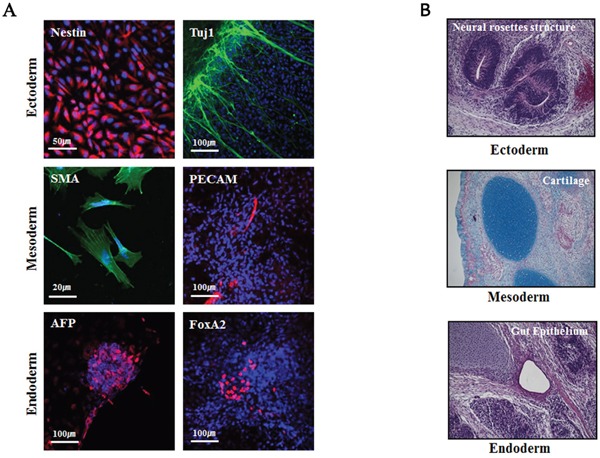
Pluripotency analysis of iPSCs **(A)** Immunostaining of representative markers of the three germ layers for the analysis of pluripotency following *in vitro* differentiation of Glis3-iPSCs. The iPSCs spontaneously differentiated, and the expression levels of representative markers of the ectoderm (Nestin, Tuj1), mesoderm (smooth muscle actin (SMA), PECAM), and endoderm (a-fetoprotein (AFP), FoxA2) lineages were examined using immunocytometry. **(B)** To perform the *in vivo* pluripotency analysis, the derivatives of the three germ layers were detected in teratomas at approximately 10 weeks after iPSC administration (2 × 10^6^ cells) into NOD/SCID mice; n = 3.

To examine whether the iPSCs retained pluripotency, the cells were spontaneously differentiated and examined for the presence of cells of the three germ layers (i.e., ectoderm, mesoderm, and endoderm). Immunostaining showed the presence of cells expressing markers for the ectoderm (Nestin and Tuj1), mesoderm (smooth muscle actin (SMA) and platelet endothelial cell adhesion molecule (PECAM)), and endoderm (alpha-fetoprotein (AFP) and forkhead box protein A2 (FOXA2)) lineages (Figure 5A). Furthermore, when the Glis3-iPSCs were injected into NOD/SCID mice, teratomas formed (Figure 5B). Together, these results demonstrated that iPSCs generated in the presence of hGlis3 retained pluripotency.

### Genome-wide gene expression analysis of Glis3-iPSCs

We next performed genome-wide gene expression profile analysis of the iPSC lines.

**Figure 6:**
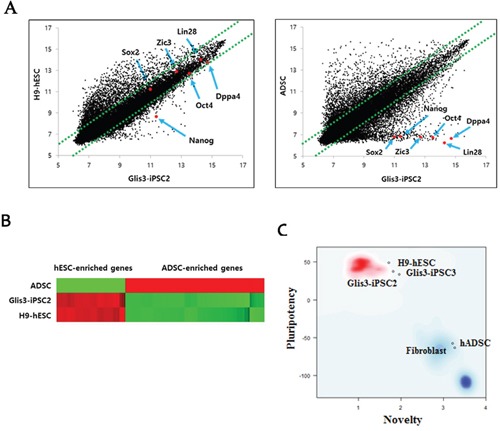
Genome-wide gene expression profiling of Glis3-iPSCs **(A)** Scatterplot analysis showed that the global gene expression pattern of Glis3-iPSCs was similar to that of H9-hESCs. However, the gene expression patterns were clearly different between Glis3-iPSCs and their parental cells, i.e., hADSCs. **(B)** Heatmap analysis indicated that the expression patterns of 80 hESC-enriched genes (Supplementary Table 5) and 96 hADSC-enriched genes (Supplementary Table 6) in Glis3-iPSCs were similar to those in H9-hESCs but different from those in hADSCs. **(C)** PluriTest, a gene expression-based bioinformatics diagnostic test for evaluating pluripotency [18], showed that the two Glis3-iPSC lines (Glis3-iPSC2 and Glis3-iPSC3) were indeed fully reprogrammed pluripotent stem cells.

Our results showed that the gene expression profile of Glis3-iPSC2 cells was close to that of H9-hESC cells. However, a significant difference in the gene expression pattern was detected between the Glis3-iPSC2 cell line and hADSCs (Figure 6). A scatter plot was created to compare the gene expression patterns of the Glis3-iPSC2 cell line and hESCs, and the results showed several representative pluripotency markers that were positioned near the diagonal line (Figure 6A). The heatmap data also demonstrated that the global gene expression profile of Glis3-iPSCs was similar to that of H9-hESCs, whereas a significantly different gene expression profile was apparent between the Glis3-iPSCs and hADSCs (Figure 6B). PluriTest, a gene expression-based bioinformatics diagnostic test for evaluating pluripotency [18], showed that the Glis3-iPSCs met the criteria for fully reprogrammed cells (Figure 6C). Taken together, the genome-wide gene expression pattern of the Glis3-iPSCs was similar to that of hESCs but very different from that of hADSCs, which were the cells from which Glis3-iPSCs originated.

## DISCUSSION

Since the first generation of Yamanaka 4 factor-mediated generation of iPSCs, many scientists have become interested in unveiling genes implicated in cellular reprogramming. In a recent study, hGlis1 was reported to promote the reprogramming of both mouse and human fibroblasts [15].

Here, we showed that another hGlis family protein, hGlis3, also retains the ability to significantly enhance reprogramming, similar to hGlis1. However, intriguingly, hGlis2 displayed a negative effect on reprogramming. With an amino acid sequence comparison, we found significant homologies among the DNA-binding domains of the hGlis proteins. Specifically, approximately 94% homology exists between the hGlis1 and hGlis3 proteins. Therefore, it is tempting to speculate that both the hGlis1 and hGlis3 proteins bind and regulate many common target genes. Based on the recently reported reprogramming activity of hGlis1, we sought to examine whether the other hGlis family proteins also retain reprogramming activity.

We found that both variants of hGlis3, which share high homology with hGlis1 in the DNA-binding domain (approximately 94%), also promote Yamanaka 4 factor (OSKM)-mediated iPSC generation as efficiently as hGlis1. In contrast, hGlis2 remarkably reduced the number of iPSC colonies formed. At this point, it remains unclear how Glis2 protein downregulates the reprogramming process. The percentage of fully reprogrammed iPSC colonies among the total colonies was also significantly reduced when Glis2 was added, further supporting the unfavorable role of this transcription factor in complete reprogramming. A recent report claimed that Glis2 is an epigenetically defined hESC biomarker that displays a conserved hESC-specific cytosine-phosphate-guanine island (CGI) methylation pattern [19]. Furthermore, the knockdown of Glis2 with siRNA induced the downregulation of pluripotency genes, such as *OCT4, NANOG* and *SOX2*, and initiated the differentiation of hESCs [19]. However, in contradiction to these observations, Pells et al. showed that overexpression of Glis2 significantly reduced the reprogramming efficiency [19], which is consistent with our result. Therefore, further efforts are needed to understand the role of Glis2 in cellular reprogramming.

In conclusion, we show that Glis family proteins are implicated in the reprogramming of somatic cells, either positively (Glis1 and 3) or negatively (Glis2). Understanding the detailed mechanisms for the effects of these Glis proteins will provide valuable insight into the process of cellular reprogramming.

## MATERIALS AND METHODS

### Plasmids

A pMXs retroviral vector containing hGlis1 cDNA was obtained from Addgene (catalog number: Plasmid #30166) (Cambridge, MA, USA). Both hGlis2 and hGlis3 (v1 and v2) cDNAs were PCR-amplified from total human cDNAs, which were generated using human dermal fibroblasts (hDFs). Each cDNA was subcloned into the yT&A cloning vector (RBC Bioscience, Taipei, Taiwan) and was sequenced (Cosmo Genetech, Seoul, Korea). The cDNAs were cut from the TA vector using EcoRI (New England Biolabs, Beverly, MA, USA) and were inserted into the EcoRI site of the retroviral vector pMXs.

### Cell culture

Human adipose derived stromal cells (hADSCs) were purchased from ScienCell (Carlsbad, CA, USA) and used for the generation of iPSCs in this study. Human ESCs (WiCell Inc., Madison, Wisconsin, USA) were used under the approval of the Institutional Review Board of CHA University.

HADSCs were cultured in alpha-MEM (WelGENE, Daegu, Korea) supplemented with 10% fetal bovine serum (FBS), 2 mM L-glutamine, and 1X penicillin/streptomycin (P/S) (all from Invitrogen, Carlsbad, CA, USA).

Human iPSCs were co-cultured on a feeder cell layer of mitomycin C (10 μg/ml) (Sigma-Aldrich, St. Louis, MO, USA)-treated STO cells (ATCC, Manassas, VA, USA) in DMEM/F12 supplemented with 20% KnockOut™ Serum Replacement, 1X non-essential amino acids (NEAA), 0.1 mM β-mercaptoethanol, 2 mM L-glutamine, 1X P/S (all from Invitrogen), and 10 ng/ml basic fibroblast growth factor (bFGF) (CHA Biotech Co., Daejeon, Korea),

### Retroviral production

293FT cells were plated at 70-80% confluency in 60-mm culture dishes (NUNC, Roskilde, Denmark) and were transfected the next day with a mixture of pMXs-Oct4, -Sox2, -Klf4, and -c-Myc along with an additional pMXs-based plasmid containing each of the four human Glis family genes (hGlis1, hGlis2, hGlisv1, and hGlis3v2). Retroviral particles were produced when two other plasmids, pGag/Pol and pVSV-G, were co-transfected with the pMXs vectors. The transfections were performed with the Convoy™ Transfection Reagent (ACTGene Inc., Piscataway, NJ, USA) according to the manufacturer's instructions.

Forty-eight hours after transfection, each supernatant was collected, filtered through a 0.45-μm syringe filter (Millipore, Billerica, MA, USA), and then subjected to ultracentrifugation (70,000 x *g*, 4°C, 120 min) (Beckman Coulter Inc., Fullerton, CA, USA) to concentrate the virus particles. The viruses were resuspended in an appropriate volume of cell culture medium, titrated, and then transduced into cells with the addition of protamine sulfate (5 μg/ml) (Sigma-Aldrich).

### Human iPSC derivation

One day before viral transduction, 5 × 10^4^ hADSCs were seeded in each well of a 6-well plate (NUNC) containing alpha-MEM supplemented with 10% FBS, 2 mM L-glutamine, and 1X P/S (all from Invitrogen). Each retrovirus was transduced at a multiplicity of infection (MOI) of 7. The medium was changed the next day, and the retroviral-infected cells were cultured for 4 additional days, transferred onto a mitomycin C-treated STO feeder layer or vitronectin (5 μg/ml) (BD Bioscience, San Jose, CA, USA)-coated 100 mm dishes (NUNC), and then further cultured in alpha-MEM supplemented with 10% FBS, 2 mM L-glutamine, and 1X P/S (all from Invitrogen). After 2 days, the medium was replaced with iPSC medium (DMEM/F12 supplemented with 20% KnockOut™ Serum Replacement, 1X NEAA, 0.1 mM β-mercaptoethanol, 2 mM L-glutamine, 1X P/S (all from Invitrogen), and bFGF (10 ng/ml) (CHA Biotech)). Individual embryonic stem cell (ESC)-like colonies were mechanically picked approximately 21-23 days after transduction and were transferred to a well of 4-well plates (NUNC) for clonal expansion.

### RNA isolation and quantitative reverse transcriptase-polymerase chain reaction (qRT-PCR)

Total RNA was isolated from various cells using the NucleoSpin RNA II Kit (MACHEREY-NAGEL GmbH & Co. KG, Duren, Germany) according to the manufacturer's protocol. One microgram of RNA was reverse transcribed to complementary DNA with the ReverTra Ace qPCR RT Kit (Toyobo, Osaka, Japan) and was subjected to qRT-PCR using the Power SYBR^®^ Green PCR Master Mix (Applied Biosystems, Foster City, CA, USA). The PCR was performed using the StepOnePlus™ Real-Time PCR System (Applied Biosystems) under the following conditions: the denaturation step was performed at 95°C for 15 sec; the annealing step was performed at 55-63°C for 30 sec; and the polymerization step was performed at 72°C for 30 sec. These steps were cycled 40 times. Human β-actin, a housekeeping gene, was used for the normalization of the relative expression of target genes. The primer sequences used in these experiments are listed in Supplementary Table 1 and Supplementary Table 3.

### Immunostaining

To examine the expression of the three germ layer markers, the cells were fixed with 4% paraformaldehyde (Sigma-Aldrich) and blocked with 5% bovine serum albumin (BSA; BOVOGEN, Essendon, Australia) in 1X PBS-T (0.2% Triton X-100) for 1 hour at room temperature (RT). The primary antibodies were diluted in 1% BSA/1X PBS-T and were added to the samples for 1-2 hours at RT. The samples were then incubated with the secondary antibodies (either Alexa 488- or 594-conjugated antibody (Invitrogen)) for 40-60 min at RT. DAPI (10 μg/ml) (Sigma-Aldrich) was used to stain nuclei. The samples were analyzed with a Zeiss LSM510 confocal microscope (Carl Zeiss, Oberkochen, Germany). The antibodies used in this study are listed in Supplementary Table 2.

### Differentiation *in vitro*

For spontaneous differentiation, the iPSC colonies were fragmented and transferred to a petri dish (SPL Lifesciences, Pochon, Korea) containing an embryoid body (EB) culture medium (DMEM/F12, 10% KnockOut™ Serum Replacement, 1% NEAA, 1X P/S and 0.1 mM β-mercaptoethanol (all from Invitrogen)). The resulting EBs were cultured for 5-10 days in suspension and were transferred onto matrigel-coated slides for 15 days of adherent culture in differentiation medium (DMEM/F12, 1% NEAA, 1X P/S, 0.1 mM β-mercaptoethanol, and 10% FBS (all from Invitrogen)). The cells were immunostained with representative markers of the three germ lineages and were observed under a TE2000U fluorescence microscope (Nikon Corporation, Tokyo, Japan).

### Teratoma formation

For teratoma formation, approximately 2 × 10^6^ cells were injected into the muscle of a NOD/SCID mouse (n=3). Teratomas were dissected approximately 10 weeks later and were subjected to hematoxylin and eosin staining for histological analysis.

### DNA fingerprinting

To confirm the origin of the iPSC lines, genomic DNA from both the iPSCs and the hADSCs was prepared, and a DNA fingerprinting analysis was performed at the Core Facility of Humanpass Inc. (Humanpass Inc., Seoul, Korea).

### Karyotyping

G-banding karyotype analyses of the hESCs, iPSCs, and hADSCs were performed at the Samkwang Medical Laboratories (Smlab, Seoul, Korea).

### DNA microarray analysis

To perform genome-wide gene expression profile experiment, total RNA was isolated using the NucleoSpin RNA II kit (MACHEREY-NAGEL GmbH) following the manufacturer's protocol, and 2 μg of total RNA was utilized for the analysis using the Illumina array (Illumina, San Diego, CA, USA) at Macrogen (Macrogen, Seoul, Korea).

## SUPPLEMENTARY MATERIALS FIGURES AND TABLES




