# Supplementary material for: Glis family proteins are differentially implicated in the cellular reprogramming of human somatic cells
Source: Oncotarget. 2017 Aug 18;8(44):77041–9. doi: 10.18632/oncotarget.20334 (PMC5652762; doi:10.18632/oncotarget.20334)
Supplement: Supplementary file 1 [file oncotarget-08-77041-s001.pdf]

Glis family proteins are differentially implicated in the cellular reprogramming of human somatic cells

SUPPLEMENTARY MATERIALS

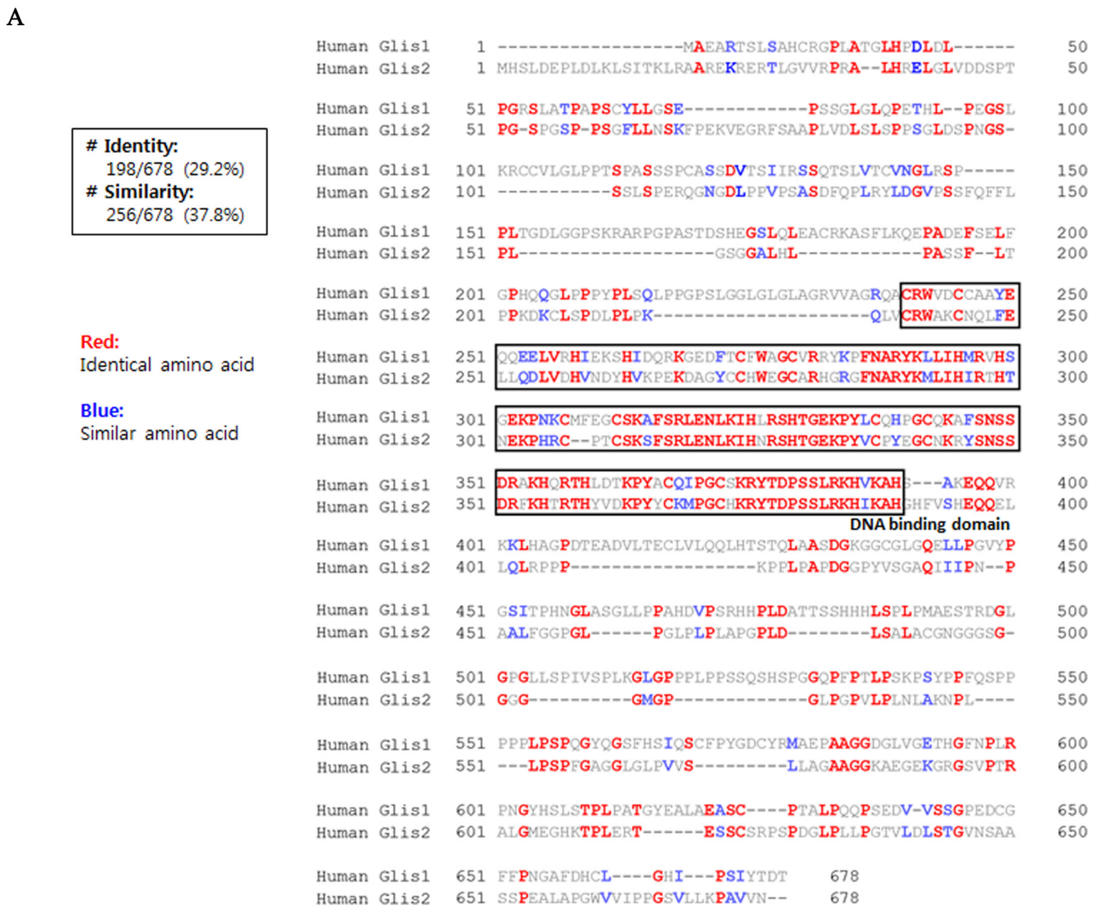

Supplementary Figure 1: The comparison of amino acid sequences between hGlis1 and hGlis2, and between hGlis1 and hGlisv2. (A) Amino acid sequence alignment between hGlis1 and hGlis2.

B

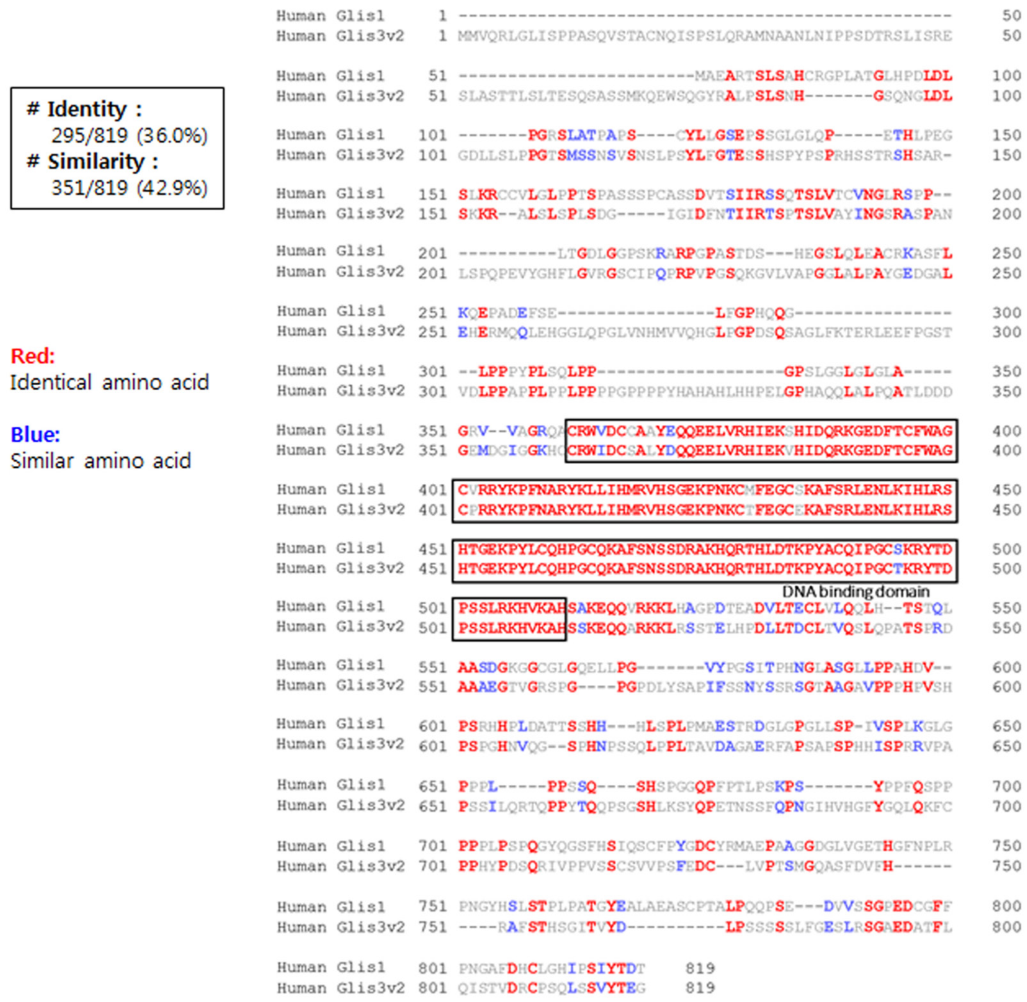

Supplementary Figure 1 (*Continued*): (B) Amino acid sequence alignment between hGlis1 and hGlis3v2.

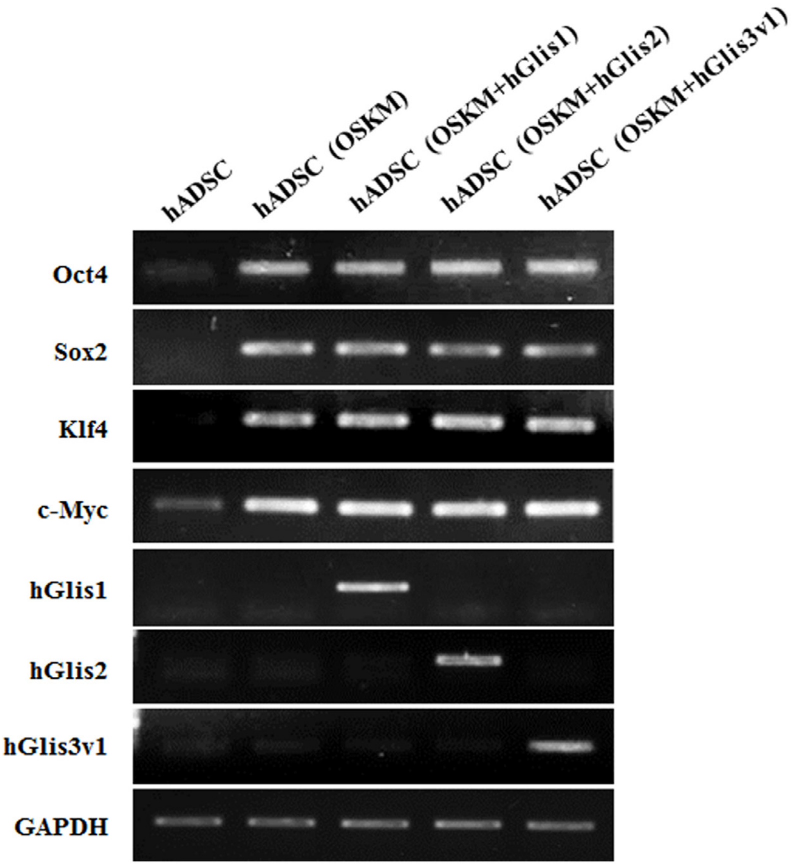

Supplementary Figure 2: RT-PCR analysis of the expression of several reprogramming genes and Glis family genes. hADSCs were transduced with retroviral vectors carrying several reprogramming genes and Glis family genes, and the expression levels of the genes were examined 70 hours after transduction.

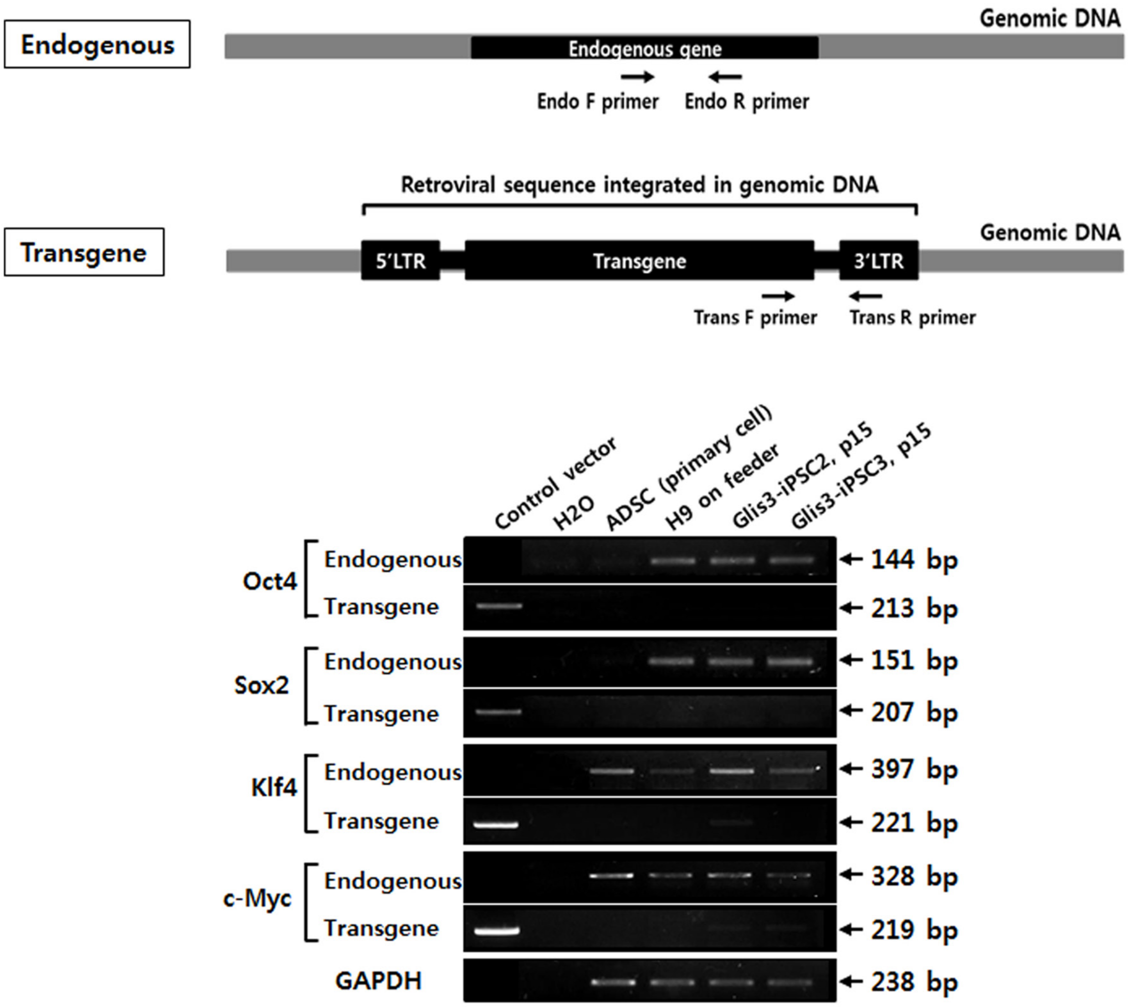

Supplementary Figure 3: RT-PCR shows the expression of exogenous and endogenous reprogramming genes in hADSCs, hESCs, and Glis3-iPSCs.

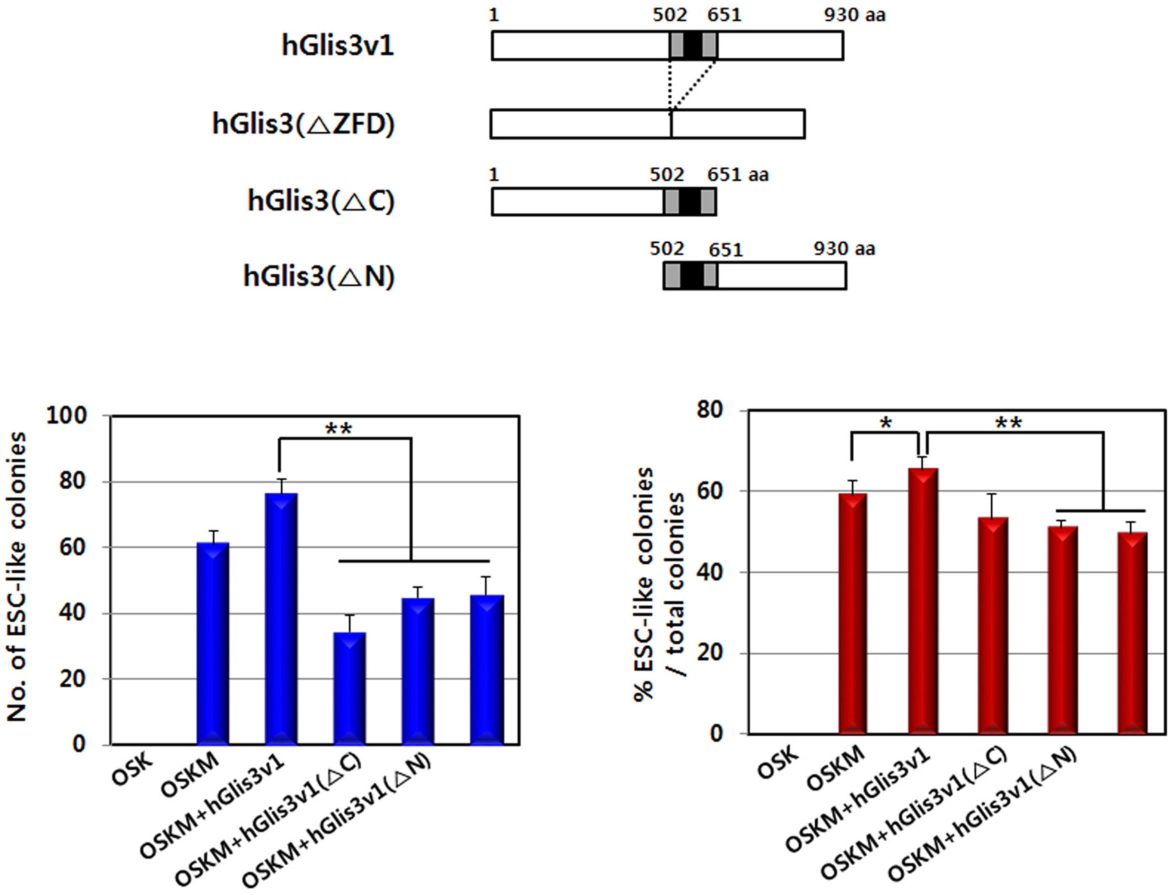

Supplementary Figure 4: Gene constructs for several truncated forms of hGlis3 proteins were generated and examined for their activities in promoting reprogramming efficiency. n=3, *p<0.05 **p<0.01.

Supplementary Table 1: List of the primer pairs used for the qRT-PCR and bisulfite sequencing analyses

Experiments	Gene	Forward primer (5' - 3')	Reverse primer (5' - 3')
qRT-PCR	Human Glis1	CACCAATAGTCAGCCCCCTG	GGCTCTGGAAGGGTGGGTAG
	Human Glis2	CATCCTGCGGACAGTTGTGG	CCAGCCATGAATGACCTAGC
	Human Glis3(v1+v2)	GAAGTGCCAGATGTGAGAAC	CAGACAGATGAGTGGATATC
	Human Glis3v1	CATCACATTCCTGCCATCCG	CGTTGTTCTGAGGAGCCATC
	Human β-actin	ATCTGGCACCACACCTTCTAC	GCGTACAGGGTAGCACAGC
	Human Oct4	AGAAGAGGATCACCCTGGGAT	AGAACCACACTCGGACCACAT
	Human Nanog	CCTGAAGACGTGTGAAGATGAG	GCTCATTAGGCTCCACCATAC
	Human Sox2	AGAAGAGGAGAGAGAAAGAAAGGGAGAGA	GAGAGAGGCAAACTGGAATCAGGATCAAA
	Human DNMT3B	GCTCACAGGGCCCGATACTT	GCAGTCCTGCAGCTCGAGTTTA
	Human Zic3	CAAAGTGTGCGACAAGTCCTACA	GGGAGGAATCTGACCCTTGAG
	Human Rex1	CAGATCCTAAACAGCTCGCAG	GCGTACGCAAATTAAAGTCCA
	Human NCAM	CACTATCTGGTCAGGTACCG	CCGCCTTGGATTTTCCTTGC
	Human Nestin	GAGAGCGAGCAGGAGGAGTT	GGAGTGGAGTCTGGAAGGGT
	Human Pax6	GCTTCACCATGGCAAATAACC	GGCAGCATGCAGGAGTATGA
	Human FoxF1	CACCAGAACAGCCACAAC	GCCATGGCGTTGAAAGAG
	Human Hand1	CACACTTGGATCGCACGT	GACAGTCCCTCCTTCTTG
	Human Gata2	CAGACGACAACCACCACCTTATG	TGGTCAGTGGCCTGTTAACATTG
	Human AFP	TTGGGCTGCTCGCTATG	TTTGTAACTGTTGCTGCCTTTG
	Human Gata6	TCCAGAAGCCAGGACTAGGA	TCCATGAACGCACATGAAAT
Bisulfite sequencing	Human Oct4	GGATGTTATTAAGATGAAGATAGTTGG	CCTAAACTCCCCTTCAAAATCTATT
	Human Nanog	AGAGATAGGAGGGTAAGTTTTTTTT	ACTCCCACACAAACTAACTTTTATTC

Supplementary Table 2: List of the antibodies used in this study

antibody (Host)	Company	Cat. No	Dilution
Oct4 (Mouse)	Santa Cruz	sc-5279	1:500
Sox2 (Rabbit)	Cell signaling	3579	1:500
SSEA4 (Mouse)	Millipore	MAB4304	1:500
Tra1-60 (Mouse)	Millipore	MAB4360	1:500
Tra1-81 (Mouse)	Millipore	MAB4381	1:500
Nestin (Mouse)	Millipore	MAB5326	1:200
Tuj1 (Mouse)	Covance	MMS-435P	1:200
SMA (Mouse)	AbFrontier	YF-PA23164	1:200
PECAM (Mouse)	Millipore	MAB1393	1:200
AFP (Mouse)	Abcam	Ab3980	1:200
FoxA2 (Mouse)	AbFrontier	YF-MA10439	1:200
Alexa Fluor 594 (Mouse)	Invitrogen	A-11005	1:500
Alexa Fluor 594 (Rabbit)	Invitrogen	A-11012	1:500
Alexa Fluor 488 (Mouse)	Invitrogen	A-11001	1:500
Alexa Fluor 488 (Rabbit)	Invitrogen	A-11008	1:500

Supplementary Table 3: List of the primer pairs used for RT-PCR analysis to distinguish the endogenous and transgene expression of the four Yamanaka reprogramming genes

Experiments	Gene	Forward primer (5' - 3')	Reverse primer (5' - 3')
**Semiquantitative RT-PCR**	Endogenous Oct4	GACAGGGGGAGGGGAGGAGCTAGG	CTTCCCTCCAACCAGTTGCCCCAAAC
	Endogenous Sox2	GGGAAATGGGAGGGGTGCAAAAGAGG	TTGCGTGAGTGTGGATGGGATTGGTG
	Endogenous Klf4	ACGATCCTGGCCCCGGAAAAGGACC	TGATTGTAGTGCTTTCTGGCTGGGCTCC
	Endogenous c-Myc	GCGTCCTGGGAAGGGAGATCCGGAGC	TTGAGGGGCATCGTCGCGGGAGGCTG
	Transgene Oct4	CCCCAGGGCCCCATTTTGGTACC	ATTTTATCGTCGACCACTGTGCTG
	Transgene Sox2	GGCACCCCTGGCATGGCTCTTGGCTC	ATTTTATCGTCGACCACTGTGCTG
	Transgene Klf4	ACGATCCTGGCCCCGGAAAAGGACC	ATTTTATCGTCGACCACTGTGCTG
	Transgene c-Myc	CAACAACCCAAAATGCACCAGCCCCAG	ATTTTATCGTCGACCACTGTGCTG

Supplementary Table 4: DNA fingerprint analysis

Locus/sample	ADSC	Glis3-iPSC2(OSKM+hGlis3)
D8S1179	10, 13	10, 13
D21S11	31, 33.2	31, 33.2
D7S820	9, 11	9, 11
CSF1PO	11, 11	11, 11
D3S1358	15, 17	15, 17
TH01	7, 9	7, 9
D13S317	8, 11	8, 11
D16S539	9, 10	9, 10
D2S1338	20, 20	20, 20
D19S433	14, 14	14, 14
vWA	18, 18	18, 18
TPOX	8, 11	8, 11
D18S51	13, 16	13, 16
D5S818	11, 11	11, 11
FGA	22, 25	22, 25

Supplementary Table 5: List of the hESC-enriched genes

See Supplementary File 1

Supplementary Table 6: List of the hADSC-enriched genes

See Supplementary File 1
