# Supplementary material for: Glis family proteins are differentially implicated in the cellular reprogramming of human somatic cells
Source: Oncotarget. 2017 Aug 18;8(44):77041–9. doi: 10.18632/oncotarget.20334 (PMC5652762; doi:10.18632/oncotarget.20334)
Supplement: Supplementary file 2 [file oncotarget-08-77041-s002.docx]

**Supplementary Table 5**

| **No.** | **hESC-enriched genes** | **Genebank** |
| --- | --- | --- |
| 1 | CD24 | NM_013230.2 |
| 2 | TUBB2B | NM_178012.3 |
| 3 | LOC642559 | XR_016333.1 |
| 4 | PODXL | NM_001018111.1 |
| 5 | DNMT3B | NM_006892.3 |
| 6 | L1TD1 | NM_019079.2 |
| 7 | GJA1 | NM_000165.3 |
| 8 | LIN28 | NM_024674.4 |
| 9 | DNMT3B | NM_006892.3 |
| 10 | RBPMS2 | NM_194272.1 |
| 11 | POU5F1P1 | NR_002304.1 |
| 12 | MT1G | NM_005950.1 |
| 13 | LOC100132564 | XM_001713808.1 |
| 14 | DPPA4 | NM_018189.3 |
| 15 | CDH1 | NM_004360.2 |
| 16 | LOC643272 | XM_926633.1 |
| 17 | MYCN | NM_005378.4 |
| 18 | TACSTD1 | NM_002354.1 |
| 19 | LOC646688 | XR_038043.1 |
| 20 | FRAT2 | NM_012083.2 |
| 21 | MARCKSL1 | NM_023009.4 |
| 22 | SEPHS1 | NM_012247.3 |
| 23 | ZFP42 | NM_174900.3 |
| 24 | TERF1 | NM_017489.1 |
| 25 | ALPL | NM_000478.3 |
| 26 | JARID2 | NM_004973.2 |
| 27 | FABP5 | NM_001444.1 |
| 28 | AIF1L | NM_031426.2 |
| 29 | PCNA | NM_182649.1 |
| 30 | LAMA5 | NM_005560.3 |
| 31 | GLDC | NM_000170.2 |
| 32 | FABP5L2 | XM_001721172.1 |
| 33 | FLJ40504 | NM_173624.1 |
| 34 | SPINT2 | NM_021102.2 |
| 35 | ACTA1 | NM_001100.3 |
| **No.** | **hESC-enriched genes** | **Genebank** |
| 36 | EPCAM | NM_002354.2 |
| 37 | SEMA6A | NM_020796.3 |
| 38 | ZIC3 | NM_003413.2 |
| 39 | CGNL1 | NM_032866.3 |
| 40 | CDCA7 | NM_031942.4 |
| 41 | GPC4 | NM_001448.2 |
| 42 | LOC729769 | XM_001131246.2 |
| 43 | ZSCAN10 | NM_032805.1 |
| 44 | DBNDD1 | NM_001042610.1 |
| 45 | DSG2 | NM_001943.3 |
| 46 | SLC7A3 | NM_032803.4 |
| 47 | SALL4 | NM_020436.2 |
| 48 | LIN28B | NM_001004317.2 |
| 49 | GPM6B | NM_001001995.1 |
| 50 | APOE | NM_000041.2 |
| 51 | AXIN2 | NM_004655.2 |
| 52 | CBX2 | NM_005189.1 |
| 53 | KIF1A | NM_004321.4 |
| 54 | PFAS | NM_012393.1 |
| 55 | NTS | NM_006183.3 |
| 56 | CTSC | NM_001814.2 |
| 57 | FAM46B | NM_052943.2 |
| 58 | KRT8 | NM_002273.2 |
| 59 | DIAPH2 | NM_006729.3 |
| 60 | CXADR | NM_001338.3 |
| 61 | NFE2L3 | NM_004289.5 |
| 62 | FGFR3 | NM_022965.1 |
| 63 | MCM3 | NM_002388.3 |
| 64 | SCNN1A | NM_001038.4 |
| 65 | MT1F | NM_005949.2 |
| 66 | LOC642956 | XM_938166.3 |
| 67 | UCA1 | NR_015379.2 |
| 68 | ST6GAL1 | NM_003032.2 |
| 69 | PDPN | NM_001006625.1 |
| 70 | ROD1 | NM_005156.4 |
| 71 | GAL | NM_015973.3 |
| **No.** | **hESC-enriched genes** | **Genebank** |
| 72 | PROM1 | NM_006017.1 |
| 73 | MT1H | NM_005951.2 |
| 74 | CKB | NM_001823.3 |
| 75 | CRMP1 | NM_001014809.1 |
| 76 | CAMKV | NM_024046.3 |
| 77 | TNFRSF21 | NM_014452.3 |
| 78 | ARID3B | NM_006465.2 |
| 79 | RPRM | NM_019845.2 |
| 80 | CDH3 | NM_001793.3 |

**Supplementary Table 6**

| **No.** | **hESC-enriched genes** | **Genebank** |
| --- | --- | --- |
| 1 | GREM1 | NM_013372.5 |
| 2 | BGN | NM_001711.3 |
| 3 | SERPINE1 | NM_000602.1 |
| 4 | DCN | NM_133505.2 |
| 5 | LOX | NM_002317.3 |
| 6 | DAB2 | NM_001343.2 |
| 7 | FER1L3 | NM_133337.1 |
| 8 | IGFBP7 | NM_001553.1 |
| 9 | IGFBP3 | NM_001013398.1 |
| 10 | COL6A2 | NM_001849.3 |
| 11 | MYOF | NM_013451.3 |
| 12 | COL12A1 | NM_080645.2 |
| 13 | HTRA1 | NM_002775.3 |
| 14 | PDGFRB | NM_002609.3 |
| 15 | COL16A1 | NM_001856.3 |
| 16 | FRMD6 | NM_152330.2 |
| 17 | FER1L3 | NM_013451.2 |
| 18 | RCAN1 | NM_203417.1 |
| 19 | MFAP5 | NM_003480.2 |
| 20 | PRKCDBP | NM_145040.2 |
| 21 | SH3PXD2A | NM_014631.2 |
| 22 | EMP1 | NM_001423.1 |
| 23 | CSRP1 | NM_004078.1 |
| 24 | PRRX1 | NM_006902.3 |
| 25 | ITGA5 | NM_002205.2 |
| 26 | FKBP9L | NM_182827.1 |
| 27 | CD248 | NM_020404.2 |
| 28 | HOXC6 | NM_004503.3 |
| 29 | CRYAB | NM_001885.1 |
| 30 | COMT | NM_007310.1 |
| 31 | SRGN | NM_002727.2 |
| 32 | ARID5B | NM_032199.1 |
| 33 | IRX3 | NM_024336.1 |
| 34 | LOC399959 | NR_024430.1 |
| 35 | FBLN5 | NM_006329.2 |
| **No.** | **hESC-enriched genes** | **Genebank** |
| 36 | ENPP2 | NM_001040092.1 |
| 37 | DAB2 | NM_001343.1 |
| 38 | IGFBP6 | NM_002178.2 |
| 39 | ALPK2 | NM_052947.3 |
| 40 | CEBPD | NM_005195.3 |
| 41 | MGC16121 | XM_001128419.1 |
| 42 | SPOCK1 | NM_004598.3 |
| 43 | ENPP2 | NM_001040092.1 |
| 44 | PCOLCE | NM_002593.2 |
| 45 | COL6A2 | NM_001849.3 |
| 46 | GAS6 | NM_000820.1 |
| 47 | RAB32 | NM_006834.2 |
| 48 | PPME1 | NM_016147.1 |
| 42 | SPOCK1 | NM_004598.3 |
| 43 | ENPP2 | NM_001040092.1 |
| 44 | PCOLCE | NM_002593.2 |
| 45 | COL6A2 | NM_001849.3 |
| 46 | GAS6 | NM_000820.1 |
| 47 | RAB32 | NM_006834.2 |
| 48 | PPME1 | NM_016147.1 |
| 49 | SRGN | NM_002727.2 |
| 50 | PARVA | NM_018222.3 |
| 51 | GAS6 | NM_000820.1 |
| 52 | S100A16 | NM_080388.1 |
| 53 | EFEMP2 | NM_016938.2 |
| 54 | SIRPA | NM_001040023.1 |
| 55 | RHOBTB3 | NM_014899.3 |
| 56 | TLN1 | NM_006289.2 |
| 57 | NFIX | NM_002501.2 |
| 58 | MFAP4 | NM_002404.1 |
| 59 | NUPR1 | NM_001042483.1 |
| 60 | HOXC8 | NM_022658.3 |
| 61 | POSTN | NM_006475.1 |
| 62 | CD99 | NM_002414.3 |
| 63 | SH3BGRL3 | NM_031286.3 |
| 64 | TIMP3 | NM_000362.4 |
| **No.** | **hESC-enriched genes** | **Genebank** |
| 65 | TSPO | NM_007311.3 |
| 66 | KCTD20 | NM_173562.3 |
| 67 | KCNK2 | NM_001017425.2 |
| 68 | COL8A1 | NM_020351.2 |
| 69 | FAP | NM_004460.2 |
| 70 | ITGA11 | NM_001004439.1 |
| 71 | KIAA1199 | NM_018689.1 |
| 72 | MME | NM_000902.3 |
| 73 | TRIB3 | NM_021158.3 |
| 74 | RFTN1 | NM_015150.1 |
| 75 | EMP3 | NM_001425.1 |
| 76 | MXRA7 | NM_001008528.1 |
| 77 | PSG5 | NM_002781.2 |
| 78 | C1orf198 | NM_032800.1 |
| 79 | GSTK1 | NM_015917.1 |
| 80 | TP53I3 | NM_147184.1 |
| 81 | RPS6KA2 | NM_001006932.1 |
| 82 | FRMD6 | NM_152330.3 |
| 83 | THRA | NM_003250.4 |
| 84 | CYBRD1 | NM_024843.2 |
| 85 | SELM | NM_080430.2 |
| 86 | AXL | NM_021913.2 |
| 87 | NRP1 | NM_003873.4 |
| 88 | CYBRD1 | NM_024843.2 |
| 89 | KLF2 | NM_016270.2 |
| 90 | LOC375295 | XM_374020.4 |
| 91 | LPAR1 | NM_057159.2 |
| 92 | RECK | NM_021111.1 |
| 93 | OAF | NM_178507.2 |
| 94 | COL4A2 | NM_001846.2 |
| 95 | NDRG1 | NM_006096.2 |
| 96 | GLIPR1 | NM_006851.2 |
